# A Comparison of the Biological Effects of ^125^I Seeds Continuous Low-Dose-Rate Radiation and ^60^Co High-Dose-Rate Gamma Radiation on Non-Small Cell Lung Cancer Cells

**DOI:** 10.1371/journal.pone.0133728

**Published:** 2015-08-12

**Authors:** Zhongmin Wang, Zhenzhen Zhao, Jian Lu, Zhijin Chen, Aiwu Mao, Gaojun Teng, Fenju Liu

**Affiliations:** 1 Institution of Molecular Imaging, Southeast University, Nanjing, China; 2 Department of Interventional Radiology, The Third Affiliated Hospital of the Medical College Shihezi University, Xinjiang, China; 3 Department of Radiology, Ruijin Hospital Luwan Branch, Shanghai Jiao Tong University School of Medicine, Shanghai, China; 4 Department of Radiology, Renji Hospital, Shanghai Jiao Tong University School of Medicine, Shanghai, China; 5 Department of Interventional Radiology, Shanghai St. Luke’s Hospital, Shanghai, China; 6 Department of Radiobiology, School of Radiological Medicine and Protection, Soochow University, Suzhou, China; University of Nebraska Medical Center, UNITED STATES

## Abstract

**Objectives:**

To compare the biological effects of ^125^I seeds continuous low-dose-rate (CLDR) radiation and ^60^Co γ-ray high-dose-rate (HDR) radiation on non-small cell lung cancer (NSCLC) cells.

**Materials and Methods:**

A549, H1299 and BEAS-2B cells were exposed to ^125^I seeds CLDR radiation or ^60^Co γ-ray HDR radiation. The survival fraction was determined using a colony-forming assay. The cell cycle progression and apoptosis were detected by flow cytometry (FCM). The expression of the apoptosis-related proteins caspase-3, cleaved-caspase-3, PARP, cleaved-PARP, BAX and Bcl-2 were detected by western blot assay.

**Results:**

After irradiation with ^125^I seeds CLDR radiation, there was a lower survival fraction, more pronounced cell cycle arrest (G_1_ arrest and G_2_/M arrest in A549 and H1299 cells, respectively) and a higher apoptotic ratio for A549 and H1299 cells than after ^60^Co γ-ray HDR radiation. Moreover, western blot assays revealed that ^125^I seeds CLDR radiation remarkably up-regulated the expression of Bax, cleaved-caspase-3 and cleaved-PARP proteins and down-regulated the expression of Bcl-2 proteins in A549 and H1299 cells compared with ^60^Co γ-ray HDR radiation. However, there was little change in the apoptotic ratio and expression of apoptosis-related proteins in normal BEAS-2B cells receiving the same treatment.

**Conclusions:**

^125^I seeds CLDR radiation led to remarkable growth inhibition of A549 and H1299 cells compared with ^60^Co HDR γ-ray radiation; A549 cells were the most sensitive to radiation, followed by H1299 cells. In contrast, normal BEAS-2B cells were relatively radio-resistant. The imbalance of the Bcl-2/Bax ratio and the activation of caspase-3 and PARP proteins might play a key role in the anti-proliferative effects induced by ^125^I seeds CLDR radiation, although other possibilities have not been excluded and will be investigated in future studies.

## Introduction

Lung cancer is the most common cancer and the leading cause of cancer-related deaths in gender-independent populations, accounting for 14% of all cancers and 28% of all cancer-related deaths worldwide [1, 2]. However, non-small cell lung cancer (NSCLC) accounts for approximately 80–85% of all lung cancer cases, and approximately 40% of these patients are diagnosed with advanced NSCLC or medically inoperable disease with a 5-year overall survival rate of less than 15% [1, 3].

In patients who are diagnosed with advanced NSCLC or medically inoperable disease, radiation therapy is usually an important treatment option; this therapy includes ^60^Co γ-ray high-dose-rate (HDR) radiation and ^125^I seeds continuous low-dose-rate (CLDR) radiation. Although external radiotherapy is still one of the main forms of cancer therapy for a wide variety of malignant human cancers, it has severe side effects on the surrounding healthy tissue. ^125^I seeds CLDR radiation offers several potential advantages over external radiotherapy, such as a localized dose distribution, sparing of normal tissue, minimal invasiveness, few complications, excellent palliation of pain and local control [4]. Consequently, ^125^I seeds CLDR radiation has gradually been used in the local treatment of patients with advanced and inoperable prostate cancer, lung cancer, pancreatic cancer, colorectal cancer and esophageal cancer [5–9].

Although many clinical trials have reported that ^125^I seeds CLDR radiation is a feasible adjuvant procedure to control local symptoms and prolong survival in advanced NSCLC, few studies have demonstrated the difference in the biological effects between ^125^I seeds CLDR radiation and ^60^Co HDR γ-ray radiation on NSCLC cells or the difference in the radiosensitivitie of NSCLC cells.

It is well known that tumor cells are characterized by uncontrolled proliferation and reduced apoptosis. To maintain genomic integrity, several DNA repair signaling pathways and cell cycle checkpoint controls are activated in response to radiation-induced damage. Cells would undergo apoptosis or death were the DNA damage not repaired or were it to accumulate sufficiently [10]. Apoptosis is a major mechanism in IR-induced cell death and most commonly occurs through the mitochondria-dependent intrinsic pathway, which involves a number of apoptosis-related genes such as Bax, Bcl-2, caspase-3 and PARP [11]. Bcl-2 and Bax are one of the most important gene pairs regulating apoptosis, and their expression is relatively stable under normal circumstances. When the level of Bcl-2 protein is increased, the activation of caspase-3 protein is inhibited. In contrast, increases in Bax protein expression promote the activation of caspase-3 protein and induce cell apoptosis [11, 12]. Caspase-3 is the most important executioner protein, and its activation results in the cleavage of PARP, which is related to DNA damage repair, and eventually apoptosis [13, 14].

To compare the radiobiological effects of ^125^I seeds CLDR radiation and ^60^Co HDR γ-ray radiation, we studied these effects in the most common pathological types of lung adenocarcinoma cells (A549 and H1299) and in human normal bronchial epithelial cells (BEAS-2B). Cell survival fraction was analyzed using clonogenic assay, cell cycle arrest induced by IR was investigated using flow cytometry (FCM), and the protein expression levels of Bcl-2, BAX, Cleaved-caspase-3 and Cleaved-PARP were detected using Western blots.

## Materials and Methods

### Cell culture and radiation conditions

In the present study, the human lung adenocarcinoma cell lines A549 and H1299 and the normal human bronchial epithelial cell line BEAS-2B were a gift from the Radiation Biological Laboratory of Soochow University (Suzhou, China). The cells were maintained in Dulbecco’s-modified Eagle’s medium (DMEM) supplemented with FBS (10%), penicillin (100 U/ml), and streptomycin (100 g/ml) in a 37°C humidified incubator with 5% CO_2_.

In this investigation, we used an in-house ^125^I seeds radiation model for CLDR [15, 16, 4], the model is constructed from polystyrene materials, and comprises a lower seed plaque layer and an upper cell culture plaque layer. In the seed plaque layer, 14 seeds were equally spaced within recesses (4.5 mm × 0.8 mm) around a 35-mm diameter (D) circumference. Thus a 6×35-mm D circumference could contain 84 seeds, and a 6×35-mm cell culture dish could be placed on the cell culture plaque layer. The height (H) between the seed plaque and the bottom of the cell culture dish was 12 mm, affording a D/H ratio of 2.9. During the CLDR, the ^125^I radiation model was always placed in a lead box in the incubator; ventholes were included around the lead box to allow the CO_2_ needed for cell growth to enter and leave the lead box, which was used to prevent radioactive leaks. Model BT-125-1 ^125^I seeds were purchased from the Shanghai GMS Pharmaceutical Co., LTD. (Shanghai, China). The activity of single ^125^I seeds was 2.5 mCi, and the initial dose rate was 18.32 cGy/h. The exposure times required to deliver various doses were calculated using the following formula: $D_{c}=D_{0}(1.44T_{1/2})(1-e^{-0.693t/T_{1/2}})$. The exposure times for delivering doses of 200, 400, 600 and 800 cGy of ^125^I seeds CLDR radiation at an initial dose rate of 18.32 cGy/h were 10.97, 22.00, 33.08, and 44.23 hours, respectively.

^60^Co HDR γ-ray radiation was generated using a 1.25 MeV GWXJ80 Cobalt-60 teletherapy unit (Nuclear Power Institute of China) at the Radiation Center, Soochow University (Suzhou, Jiangsu, China) [17]. The dose rate used was 0.5 Gy/min. The distance between the radiation source and the cell plane was 0.8 m. The exposure times needed to deliver doses of 200, 400, 600 and 800 cGy using ^60^Co HDR γ-ray radiation at the initial dose rate of 0.5 Gy/min were 4, 8, 12 and 16 minutes, respectively. Cells undergoing exponential growth were exposed to ^125^I seeds CLDR radiation or to ^60^Co HDR γ-ray radiation at 2, 4, 6 and 8 Gy. Control cells were subjected to the same procedure without radiation (0 Gy).

### Clonogenic survival assay

A549, H1299 and BEAS-2B cells undergoing exponential growth were trypsinized to form a single cell suspension and seeded into 35-mm culture plates at various dilutions [18] (cell numbers were decided according to irradiation dose, as follows: 200 cells per dish for the control and 2 Gy treatments, 500 cells for the 4 Gy treatment, 1,000 cells for the 6 Gy treatment and 4,000 cells for 8 the Gy treatment). After a24-h incubation, the A549, H1299 and BEAS-2B cells were exposed to ^125^I seeds CLDR radiation or to ^60^Co HDR γ-ray radiation of 0, 2, 4, 6 or 8 Gy, after which the cells were placed in a 37°C incubator and cultured for 10–14 d to form clones. Next, the colonies were fixed with methanol and stained with crystal violet. Colonies with more than 50 cells were counted as colony-forming units.

### Cell cycle analysis by flow cytometry (FCM)

A549, H1299 and BEAS-2B cells undergoing exponential growth were exposed to ^125^I seeds CLDR radiation or ^60^Co HDR γ-ray radiation of 0, 2, 4, 6 or 8 Gy. After irradiation, cells were continuously cultured for 24 h. Then, the cells were trypsinized and centrifuged at 1000 rpm for 5 min. The supernatant was discarded, and the cells were washed with cold phosphate-buffered saline (PBS) one to two times and fixed in 70% alcohol at 4°C overnight. The cells were then centrifuged again, and the fixation solution was discarded. Subsequently, the cells were washed with cold PBS one or two times and then incubated with 10 mg/ml RNase A and 500 μg/ml PI in the dark for 15–30 min at room temperature to determine the cell cycle progression by FCM.

### Apoptosis analysis by FCM

The three cell types were irradiated with ^125^I seeds or ^60^Co of 0, 2, 4, 6 or 8 Gy 24 h after plating. At 48 h after irradiation, the cells were harvested, resuspended in 500 μl binding buffer and mixed with 5 μl of Annexin V-FITC and 5 μl of PI for 15–30 min in the dark at room temperature according to the manufacturer’s instructions (Biuniquer Technology) to examine the apoptotic ratio by FCM.

### Western blotting

A549, H1299 and BEAS-2B cells undergoing exponential growth were exposed to ^125^I seeds CLDR radiation or ^60^Co HDR γ-ray radiation of 0, 4, or 8 Gy. The cells were harvested, centrifuged and washed twice with ice-cold 1×PBS 24 h after irradiation. Subsequently, total protein was extracted with lysis buffer (Beyotime, China) containing 1 mM PMSF (Beyotime, China) and 1 protease inhibitor cocktail tablet (Roche Applied Science, Mannheim, Germany) per 10 ml of solution was added. Next, the total protein was isolated. After centrifugation, the supernatant was transferred into new tubes. The protein concentrations were determined by BCA assay (Pierce Biotechnology, Rockford, IL, USA). A total volume of 20 μl of sample containing 50 μg of protein was separated by SDS–PAGE and electroblotted onto polyvinylidene fluoride (PVDF) membranes (Bio-Rad Laboratories, Hercules, CA, USA). The membranes were then blocked with 5% non-fat milk in TBS-T (20 mmol/L Tris-HCl, 150 mmol/L NaCl, and 0.1% Tween-20) for 1 h at room temperature and subsequently incubated with the appropriate primary antibodies overnight at 4°C. After being completely washed with TBST, the membranes were incubated with horseradish peroxidase-conjugated secondary antibodies for 1 h at room temperature, after which, the membranes were again washed with TBST. Then, the proteins were detected using the enhanced chemiluminescence (ECL) system with prestained markers as molecular size standards.

The primary antibodies used in our study were anti-β-actin (1:1000 dilution, 60008-1-Ig, Proteintech, USA), anti-caspase-3 (1:1000 dilution, 19677-1-AP, Proteintech, USA), anti-PARP (1:1000 dilution, 13371-1- AP, Proteintech, USA), anti-Bax (1:1000 dilution, 50599-2-Ig, Proteintech, USA) and anti-Bcl-2 (1:1000 dilution, 12789-1-AP, Proteintech, USA). The secondary antibodies used in our study were horseradish peroxidase-labeled goat anti-rat IgG (H+L) (1:2000, A0192, Beyotime Institute of Biotechnology, China) and horseradish peroxidase-labeled goat anti-rabbit IgG (H+L) (1:2000, A0208, Beyotime Institute of Biotechnology, China).

### Statistical analyses

All results are presented as the mean±standard error (SE) of three independent experiments, and each experiment used three parallel samples. Significant differences in the data of different groups were evaluated by Student’s *t* test and one-way ANOVA. Significance was set at P-values below 0.05.

## Results

### Clonogenic survival

**Fig 1:**
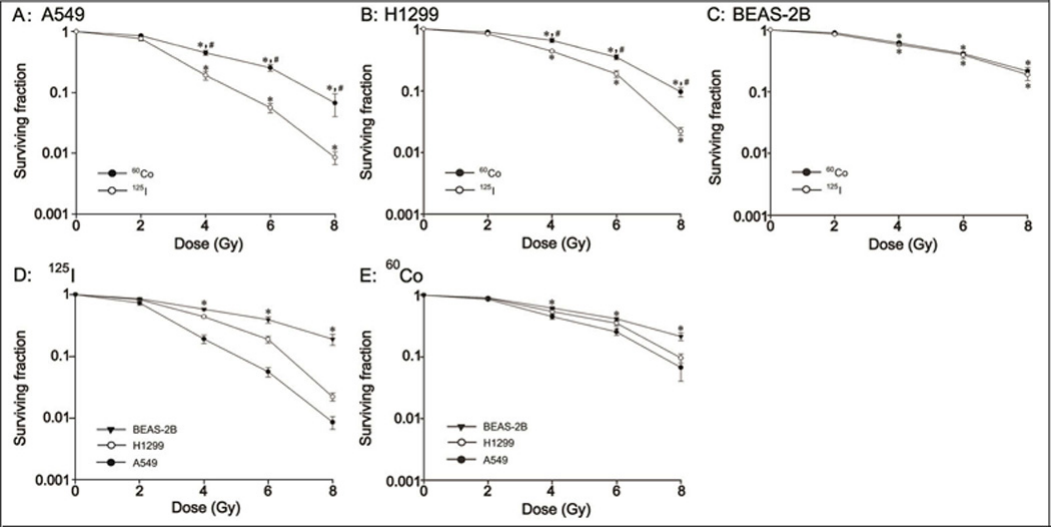
Clonogenic survival fraction of A549, H1299 and BEAS-2B cells irradiated with ^125^I seeds and ^60^Co γ-rays. A-C: the difference in the survival fraction of A549, H1299 and BEAS-2B cells between ^125^I seeds CLDR radiation and ^60^Co γ-ray HDR radiation. * indicates a significant difference (P<0.05) when comparing the two radiation treatments and control treatment, and # indicates a significant difference (P<0.05) when comparing ^125^I seeds CLDR radiation and ^60^Co γ-ray HDR radiation of the same doses. D, E: The difference in the survival fraction of the three cell lines receiving the same treatment. * indicates a significant difference (P<0.05). Bars, standard error (SE).

To examine whether the anti-proliferative effects induced by ^125^I seeds CLDR radiation were more efficient than those of ^60^Co HDR γ-ray radiation on A549, H1299, and BEAS-2B cells and to detect whether the radiosensitivities of the three cell lines were the same, we performed a clonogenic survival assay. As shown in Fig 1 (S1 Dataset), the survival fractions of A549, H1299 and BEAS-2B cells after ^125^I seeds CLDR radiation and ^60^Co HDR γ-ray radiation were significantly decreased compared with that of control cells (P<0.05). For A549 cells, the survival fraction induced by ^125^I seeds CLDR radiation was significantly lower than that of ^60^Co HDR γ-ray radiation at 4, 6 and 8 Gy (Fig 1A, P<0.05); however, the difference was not significant at 2 Gy. For H1299 cells, the survival fraction in the ^125^I seeds CLDR radiation group was also decreased significantly compared with that of the ^60^Co HDR γ-ray radiation group at 4, 6 and 8 Gy (Fig 1B, P<0.05). However, we did not observe a difference in the survival fraction between normal BEAS-2B cells treated with the two radiation sources at the same doses (Fig 1C, P>0.05). Fig 1D and 1E show that the survival fraction from lowest to highest was A549<H1299<BEAS-2B in both the ^125^I seeds CLDR-radiation group and the ^60^Co HDR γ-ray-radiation group (P<0.05).

### G_1_ arrest in A549 cells and G_2_/M arrest in H1299 and BEAS-2B cells

**Fig 2:**
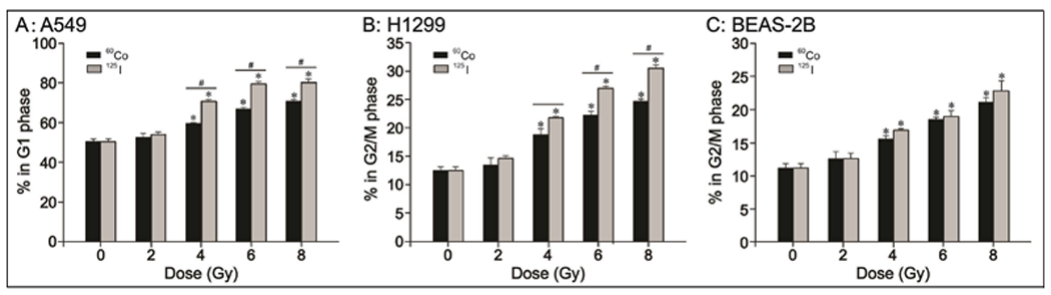
Cell cycle analysis by flow cytometry. A: G_1_ arrest of A549 cells irradiated with the two radiation sources; B, C: G_2_/M arrest of H1299 and BEAS-2B cells after irradiation with the two radiation sources. * indicates a significant difference (P<0.05) when comparing 2, 4, 6 and 8 Gy irradiation by the two radiation sources and control (0 Gy) treatment, and # indicates a significant difference (P<0.05) when comparing the two radiation treatments of the same dose. Bars, standard error (SE).

To explore whether ^125^I seeds CLDR radiation and ^60^Co HDR γ-ray radiation cause different types of cell cycle arrest, we analyzed the cell cycle distribution after irradiation with the two radiation sources at 0, 2, 4, 6 and 8 Gy. As shown in Fig 2 (S2 Dataset), we observed G_1_ arrest in A549 cells and G_2_/M arrest in H1299 and BEAS-2B cells after irradiation with ^125^I seeds and ^60^Co radiation of 4, 6 and 8 Gy compared with control cells (P<0.05); however, the difference was not significant at 2 Gy. Moreover, more A549 cells were in G_1_ phase and more H1299 cells were in G_2_/M phase after ^125^I seeds CLDR radiation than after ^60^Co HDR γ-ray radiation at 4, 6 and 8 Gy; this trend was dose-dependent (Fig 2A and 2B, P<0.05). However, there was no significant difference in G_2_/M arrest between ^125^I seeds CLDR radiation and ^60^Co HDR γ-ray radiation in normal BEAS-2B cells (Fig 2C, P>0.05). After ^125^I seeds CLDR radiation of 4 Gy, the G_1_ phase percentage of A549 cells was 70.67±0.86%, and the G_2_/M phase percentages for H1299 and BEAS-2B cells were 21.77±0.31% and 16.86±0.29%, respectively. However, only 59.59±0.41% of A549 cells were in G_1_ phase and 18.85±0.99% of H1299 cells and 15.58±0.48% of BEAS-2B cells were in G_2_/M phase after ^60^Co HDR γ-ray radiation of 4 Gy; other data are as shown in Fig 2.

### Apoptotic ratio of A549, H1299 and BEAS-2B cells

**Fig 3:**
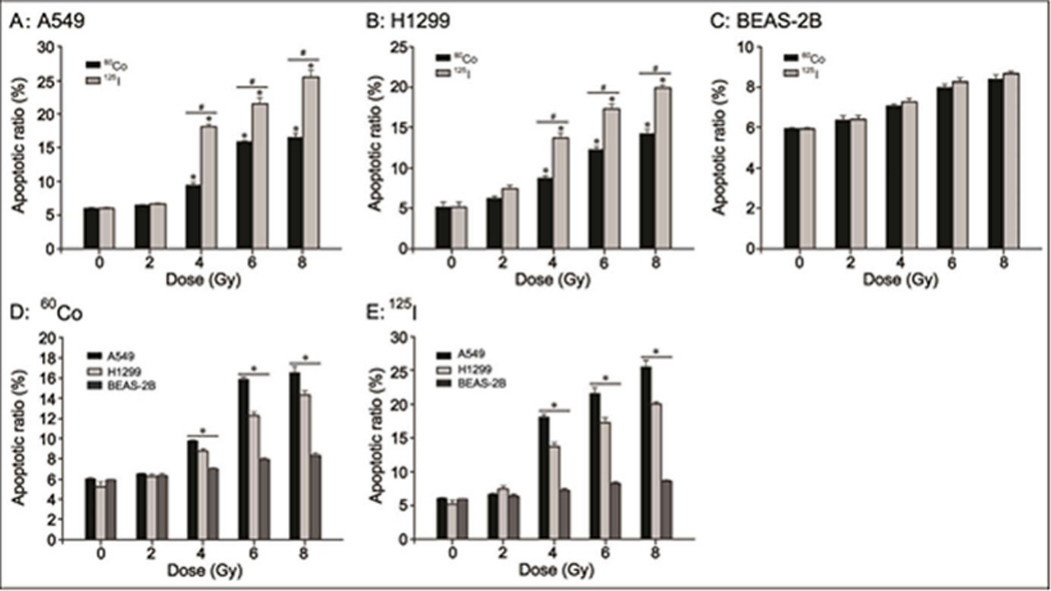
Apoptotic ratio of A549, H1299 and BEAS-2B cells irradiated with ^125^I seeds CLDR radiation and ^60^Co γ-ray HDR radiation. A-C: the difference in the apoptotic ratio of A549, H1299 and BEAS-2B cells receiving ^125^I seeds CLDR radiation and ^60^Co γ-ray HDR radiation. * indicates a significant difference (P<0.05) when comparing 2, 4, 6 and 8 Gy irradiation by the two radiation sources and control (0 Gy) treatment, and # indicates a significant difference (P<0.05) when comparing the two radiation treatments of the same doses. D, E: the difference in the apoptotic ratios of the three cell lines irradiated with ^125^I seeds and ^60^Co γ-rays. * indicates a significant difference (P<0.05). Bars, standard error (SE).

Cell apoptosis induced by IR is one of the most important effects of tumor radiotherapy. Therefore, the apoptotic ratios of A549, H1299 and BEAS-2B cells after irradiation with ^125^I seeds and ^60^Co γ-rays at 0, 2, 4, 6 and 8 Gy were examined (S3 Dataset). As shown in Fig 3A and 3B, both ^125^I seeds CLDR radiation and ^60^Co HDR γ-ray radiation led to a marked increase in the apoptotic ratio of A549 and H1299 cells compared with control treatment at 4, 6 and 8 Gy (P<0.05), while the apoptotic ratio was similar at 2 Gy between the two types of radiation treatment and the control. As expected, ^125^I seeds CLDR radiation led to a higher percentage of apoptotic A549 and H1299 cells than did ^60^Co HDR γ-ray radiation at 4, 6 and 8 Gy (Fig 3A and 3B, P<0.05). However, there was no difference in the apoptotic ratio of BEAS-2B cells receiving ^125^I seeds CLDR radiation, ^60^Co HDR γ-ray radiation or control treatment (Fig 3C, P>0.05). As shown in Fig 3D and 3E, the apoptotic ratio was A549>H1299>BEAS-2B for both the ^125^I seeds CLDR radiation group and the ^60^Co HDR γ-ray radiation group (P<0.05). The apoptotic ratios were 18.09±0.42% and 13.79±0.50% for A549 cells and H1299 cells, respectively, after ^125^I seeds CLDR radiation of 4 Gy. In contrast, the apoptotic ratios were only 9.46±0.42% and 8.79±0.22%, respectively, after ^60^Co HDR γ-ray radiation of 4 Gy; other data are as shown in Fig 3.

### The imbalance of Bcl-2 and Bax proteins

**Fig 4:**
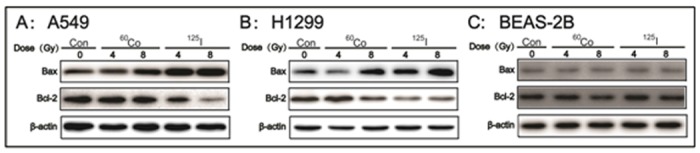
The expression of Bcl-2 and BAX proteins in A549, H1299 and BEAS-2B cells after exposure to ^125^I seeds CLDR radiation and ^60^Co γ-ray HDR radiation of 4 and 8 Gy. One representative result of three independent experiments with identical results is shown. Con: control.

The clonogenic survival assay, cell cycle analysis and cell apoptosis analysis results obtained using FCM suggest that the differences between these two radiation mehtods and the blank control group were not significant at 2 Gy irradiation; therefore, we chose 4 and 8 Gy for use in this study. It has been suggested that Bcl-2 and Bax represent one of the most important gene pairs for regulating of apoptosis [19, 20]. Therefore, Bcl-2 and Bax protein expression was detected by western blot assay. As shown in Fig 4A and 4B, both ^125^I seeds CLDR radiation and ^60^Co HDR γ-ray radiation of 4 and 8 Gy could significantl up-regulate the expression of Bax protein and down-regulate the expression of Bcl-2 protein in A549 and H1299 cells compared with control treatment. The effects induced by ^125^I seeds CLDR radiation were more obvious than those of ^60^Co HDR γ-ray radiation. As expected, there was no increase in Bcl-2 and BAX expression in BEAS-2B cells after irradiation by either ^125^I seeds CLDR radiation or ^60^Co HDR γ-ray radiation (Fig 4C).

### The activation of caspase-3 and PARP

**Fig 5:**
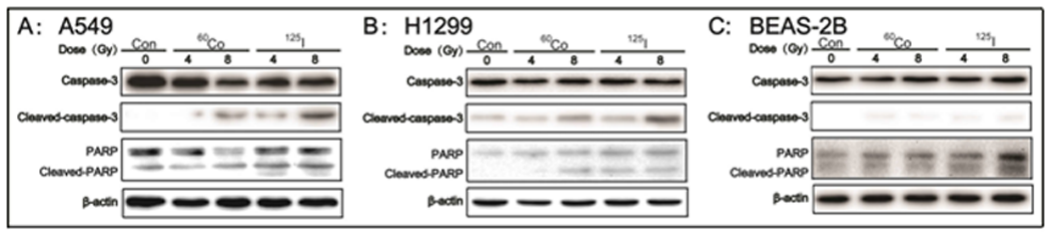
The expression of cleaved-caspase-3 and cleaved-PARP proteins in A549, H1299 and BEAS-2B cells after exposure to ^125^I seeds CLDR radiation and ^60^Co γ-ray HDR radiation of 4 and 8 Gy. One representative result of three independent experiments with identical results is shown.

Activation of caspase-3 and PARP proteins is commonly considered the biochemical hallmarks of an apoptotic event. Therefore, cleaved-caspase-3 and cleaved-PARP proteins were examined by western blot assay. As illustrated in Fig 5A and 5B, the expressions of Cleaved-caspase-3 and Cleaved-PARP proteins were up-regulated by both ^125^I seeds CLDR radiation and ^60^Co HDR γ-ray radiation. Furthermore, the expression levels of Cleaved-caspase-3 and Cleaved-PARP were much higher in A549 and H1299 cells that had been subjected to ^125^I seeds CLDR radiation than in those that had been subjected to ^60^Co HDR γ-ray radiation. However, no difference was apparent in the levels of cleaved-caspase-3 and cleaved-PARP proteins among BEAS-2B cells exposed to ^125^I seeds CLDR radiation, ^60^Co HDR γ-ray radiation and sham radiation (Fig 5C).

## Discussion

In this study, ^125^I seeds CLDR radiation led to more remarkable A549 and H1299 cell growth inhibition than did ^60^Co HDR γ-ray radiation, which is consistent with the results of other investigations [21]; however, the difference was not significant at 2 Gy for the two radiation treatments. We also saw clearly that the radiosensitivity was A549>H1299>BEAS-2B, which is in agreement with the results reported by Masahiko Nishizaki et al. [22]. Our results also suggested that BEAS-2B cells are resistant to irradiation relative to A549 and H1299 cells.

It is well known that each phase of the cell cycle contains checkpoints that allow for arrest of cell cycle progression to either activate repair mechanisms or activate the cell apoptotic cascade and cell death after DNA damage induced by IR or other factors [23, 24]. In our study, significant G_1_ arrest of A549 cells was induced more by ^125^I seeds CLDR radiation than by ^60^Co HDR γ-ray radiation at 4, 6 and 8 Gy, which is consistent with other studies that evaluated pancreatic and prostate cancer cells [25, 26]. However, we should note that there were also some differences. For instance, Junjie Wang reported that ^125^I seeds CLDR radiation led to long-term G_2_/M arrest of A549 cells at 4 Gy with an initial dose rate of 2.77 cGy/h [21], which is in contrast to the significant G_1_ arrest with an initial dose rate of 18.32 cGy/h observed in our study. The difference in the initial dose rate and a variety of other factors such as the status of cells may, at least in part, account for this difference. The specific mechanisms underlying this phenomenon are unknown. However, there was G_2_/M arrest in H1299 and BEAS-2B cells receiving the same treatment. Geyer and coworkers have reported G_1_ arrest in A549 cells and G_2_/M arrest in H1299 cells under the same irradiation conditions, and no G_1_ arrest was observed in IR-treated cells that lack p53 [27]. These results are similar to the results of the present study. Although there was significant G_2_/M arrest of normal BEAS-2B cells, the extent of G_2_/M arrest caused by the two radiation sources was similar, which is consistent with the colony-forming capacity observed in the present study.

Apoptosis, also known as programmed cell death, is the major mechanism of IR-induced cell death. To maintain genomic integrity, multiple DNA repair signaling pathways and cell cycle checkpoint controls are activated in response to radiation-induced damage, but if these repair processes fail or irreparable damage accumulates to a certain degree, cell apoptosis or cell death is induced [10]. According to our observations, ^125^I seeds CLDR radiation led to a higher percentage of apoptotic A549 and H1299 cells than did ^60^Co HDR γ-ray radiation, while the apoptotic ratio of BEAS-2B cells induced by the two radiation sources at 2, 4, 6 and 8 Gy was similar to that of control cells, which was consistent with other studies [19]. In our study, the difference in the results obtained using ^125^I seeds CLDR radiation and ^60^Co gamma ray HDR radiation might appear surprising. For x- or γ-rays, dose rate is among the main factors that determine the biological consequences of a given absorbed dose; as the dose rate is reduced and the exposure time extended, the biological effect of the given dose is generally reduced [28]. However, when the dose rate is reduced to less than 1 Gy/h, decreasing the dose rate might result in increased cell killing; this effect has been was termed an inverse dose-rate effect and low-dose hyper-radiosensitivity (HRS) [29].

In our study, the results obtained using a clonogenic survival assay, cell cycle analysis and cell apoptosis analysis using FCM suggested that the differences between the tested radiation treatment group and the blank control group were not significant at 2Gy; therefore, we focused on the potential molecular mechanisms underlying the more distinct anti-proliferative effect of ^125^I seeds CLDR radiation compared with ^60^Co HDR γ-ray radiation at 4 and 8Gy.

Apoptosis most commonly occurs through the mitochondria-dependent intrinsic pathway, which is a complex process that involves a number of apoptosis-related proteins, including caspase-3, PARP, Bax and Bcl-2 et al. [11]. The function of Bcl-2 protein, also known as pro-survival protein Bcl-2, is to inhibit apoptosis and prolong cell survival. Over-expression of Bcl-2 protein is associated with a poor response to lung cancer treatment [30]. Bax, a pro-apoptotic protein of the Bcl-2 family, plays a key role in mediating the apoptotic response [12]. The ratio of Bcl-2 to Bax is commonly considered a determinant in the initiation of apoptosis [31]. Caspase-3 protein is the most important executioner protein. The function of PARP is the routine repair of DNA damage, and PARP is the main substrate of caspase-3. Therefore, the activation of caspase-3 and PARP proteins were commonly considered the biochemical hallmarks of apoptosis [30, 32]. In our study, the imbalance between Bcl-2 protein and BAX protein and the higher levels of cleaved-caspase-3 and cleaved-PARP proteins might partly have promoted the increased apoptosis seen in A549 and H1299 cells after irradiation with ^125^I seeds compared with ^60^Co γ-rays. For a cell to survive the lethal damage of DNA DSBs that is caused by irradiation exposure must be rapidly detected, and DNA repair mechanisms must be initiated. Some researchers have demonstrated that greater cell killing is caused by the inefficient activation or a lower activation of the DNA damage sensor ataxia telangiectasia-mutated (ATM) gene in cells irradiated at low dose rate (LDR) radiation compared to high dose rate (HDR) radiation even when the DNA has already been damaged. In addition, the ATM-associated repair pathways might not be activated after irradiation by LDR radiation [33, 28]. IR is known to cause G_2_/M or G_1_ arrest and to induce apoptosis in irradiated cells [19]. However, in the present study, the two radiation treatments induced a slight decrease in the survival fraction and significant G_2_/M arrest but failed to induce significant apoptosis in BEAS-2B cells.

## Conclusions

In summary, our results demonstrate that ^125^I seeds CLDR radiation leads to more remarkable growth inhibition of A549 and H1299 cells than that of ^60^Co HDR γ-ray radiation. We also found that A549 cells were the most sensitive to radiation, followed by H1299 cells, while BEAS-2B cells were more resistant to the two radiation treatments than A549 and H1299 cells. Moreover, this study showed that an imbalance of the Bcl-2/Bax ratio and activation of caspase-3 and PARP proteins might partly account for the anti-proliferative effect induced in A549 and H1299 cells by ^125^I seeds CLDR radiation. However, this study only concentrated on apoptosis-related events and did not focus in depth on DNA damage repair and cell cycle checkpoints due to time and funding limitations. Nevertheless, our data illustrate some fundamental radiobiological effects in NSCLC cells when subjected to ^125^I seeds CLDR radiation.

## Supporting Information

S1 Dataset The survival fractions of A549, H1299 and BEAS-2B cells after ^125^I seeds CLDR radiation and ^60^Co HDR γ-ray radiation. (DOC)

S2 Dataset The cell cycle distribution after irradiation with ^125^I seeds and ^60^Co γ-rays. (%) (DOC)

S3 Dataset The apoptotic ratios of A549, H1299 and BEAS-2B cells after irradiation with ^125^I seeds and ^60^Co γ-rays. (%) (DOC)
