# Supplementary material for: A Comparison of the Biological Effects of 125I Seeds Continuous Low-Dose-Rate Radiation and 60Co High-Dose-Rate Gamma Radiation on Non-Small Cell Lung Cancer Cells
Source: PLoS One. 2015 Aug 12;10(8):e0133728. doi: 10.1371/journal.pone.0133728 (PMC4534329; doi:10.1371/journal.pone.0133728)
Supplement: S1 Dataset — (DOC) [file pone.0133728.s001.doc]

|  | 0Gy | | 2Gy | | 4Gy | | 6Gy | | 8Gy | |
| --- | --- | --- | --- | --- | --- | --- | --- | --- | --- | --- |
|  | ^60^Co | ^125^I | ^60^Co | ^125^I | ^60^Co | ^125^I | ^60^Co | ^125^I | ^60^Co | ^125^I |
| BEAS-2B | 1 | 1 | 0.90 | 0.85 | 0.62 | 0.55 | 0.41 | 0.37 | 0.20 | 0.18 |
|  | 1 | 1 | 0.88 | 0.81 | 0.64 | 0.63 | 0.37 | 0.35 | 0.27 | 0.16 |
|  | 1 | 1 | 0.91 | 0.89 | 0.58 | 0.56 | 0.45 | 0.45 | 0.17 | 0.25 |
| A549 | 1 | 1 | 0.90 | 0.76 | 0.45 | 0.17 | 0.25 | 0.05 | 0.07 | 0.01 |
|  | 1 | 1 | 0.84 | 0.65 | 0.53 | 0.17 | 0.35 | 0.07 | 0.05 | 0.01 |
|  | 1 | 1 | 0.81 | 0.86 | 0.43 | 0.23 | 0.27 | 0.04 | 0.10 | 0.01 |
| H1299 | 1 | 1 | 0.88 | 0.79 | 0.42 | 0.36 | 0.29 | 0.18 | 0.10 | 0.0.5 |
|  | 1 | 1 | 0.85 | 0.86 | 0.51 | 0.42 | 0.23 | 0.15 | 0.10 | 0.04 |
|  | 1 | 1 | 0.93 | 0.83 | 0.47 | 0.39 | 0.34 | 0.23 | 0.10 | 0.04 |
