# Supplementary material for: A Comparison of the Biological Effects of 125I Seeds Continuous Low-Dose-Rate Radiation and 60Co High-Dose-Rate Gamma Radiation on Non-Small Cell Lung Cancer Cells
Source: PLoS One. 2015 Aug 12;10(8):e0133728. doi: 10.1371/journal.pone.0133728 (PMC4534329; doi:10.1371/journal.pone.0133728)
Supplement: S2 Dataset — (DOC) [file pone.0133728.s002.doc]

|  | 0Gy | | 2Gy | | 4Gy | | 6Gy | | 8Gy | |
| --- | --- | --- | --- | --- | --- | --- | --- | --- | --- | --- |
|  | ^60^Co | ^125^I | ^60^Co | ^125^I | ^60^Co | ^125^I | ^60^Co | ^125^I | ^60^Co | ^125^I |
| BEAS-2B  (G2/M) | 12.19 | 13.19 | 12.16 | 13.32 | 14.68 | 17.35 | 18.55 | 19.58 | 22.35 | 23.11 |
|  | 10.03 | 11.53 | 14.63 | 13.56 | 15.76 | 16.34 | 17.86 | 20.11 | 20.04 | 25.30 |
|  | 11.36 | 11.36 | 11.01 | 11.11 | 16.30 | 16.89 | 19.13 | 17.12 | 21.03 | 20.03 |
| A549  (G1) | 50.47 | 49.28 | 48.55 | 56.21 | 58.79 | 70.72 | 66.65 | 81.21 | 69.63 | 83.31 |
|  | 43.68 | 54.36 | 55.68 | 53.48 | 59.86 | 72.13 | 65.41 | 79.33 | 72.02 | 79.21 |
|  | 49.63 | 47.61 | 53.31 | 52.17 | 60.13 | 69.16 | 68.32 | 78.01 | 70.51 | 78.36 |
| H1299  (G2/M) | 13.01 | 13.36 | 14.23 | 15.36 | 18.55 | 21.65 | 21.09 | 27.10 | 24.66 | 30.99 |
|  | 12.89 | 12.17 | 15.19 | 14.17 | 19.68 | 22.34 | 23.43 | 26.39 | 25.35 | 31.25 |
|  | 11.67 | 12.67 | 11.01 | 14.67 | 16.32 | 21.32 | 22.31 | 27.48 | 24.05 | 29.38 |
