# Supplementary material for: A Comparison of the Biological Effects of 125I Seeds Continuous Low-Dose-Rate Radiation and 60Co High-Dose-Rate Gamma Radiation on Non-Small Cell Lung Cancer Cells
Source: PLoS One. 2015 Aug 12;10(8):e0133728. doi: 10.1371/journal.pone.0133728 (PMC4534329; doi:10.1371/journal.pone.0133728)
Supplement: S3 Dataset — (DOC) [file pone.0133728.s003.doc]

|  | 0Gy | | 2Gy | | 4Gy | | 6Gy | | 8Gy | |
| --- | --- | --- | --- | --- | --- | --- | --- | --- | --- | --- |
|  | ^60^Co | ^125^I | ^60^Co | ^125^I | ^60^Co | ^125^I | ^60^Co | ^125^I | ^60^Co | ^125^I |
| BEAS-2B | 5.95 | 5.45 | 6.56 | 6.73 | 7.01 | 7.56 | 8.42 | 8.22 | 8.56 | 8.38 |
|  | 5.18 | 6.68 | 6.19 | 6.21 | 7.49 | 7.20 | 7.81 | 8.01 | 8.63 | 8.71 |
|  | 5.82 | 5.62 | 5.99 | 6.49 | 6.72 | 7.01 | 7.67 | 8.58 | 7.96 | 8.97 |
| A549 | 6.25 | 6.87 | 6.55 | 6.67 | 9.76 | 17.26 | 15.70 | 21.63 | 17.69 | 25.19 |
|  | 6.50 | 5.97 | 6.64 | 6.31 | 9.68 | 18.65 | 16.23 | 23.02 | 15.64 | 24.00 |
|  | 5.27 | 5.35 | 6.43 | 6.91 | 9.99 | 18.36 | 15.76 | 20.13 | 16.23 | 27.39 |
| H1299 | 5.48 | 5.38 | 6.48 | 6.87 | 8.85 | 14.68 | 12.33 | 17.35 | 15.02 | 20.11 |
|  | 5.03 | 5.28 | 6.56 | 7.28 | 9.14 | 13.74 | 11.74 | 18.37 | 14.46 | 20.43 |
|  | 5.29 | 5.21 | 5.83 | 8.21 | 8.37 | 12.96 | 12.85 | 16.22 | 13.39 | 19.38 |
